# Nanotechnology-Assisted Isolation and Analysis of Circulating Tumor Cells on Microfluidic Devices

**DOI:** 10.3390/mi11080774

**Published:** 2020-08-14

**Authors:** Jie Cheng, Yang Liu, Yang Zhao, Lina Zhang, Lingqian Zhang, Haiyang Mao, Chengjun Huang

**Affiliations:** 1 Institute of Microelectronics of Chinese Academy of Sciences, Beijing 100029, China; chengjie@ime.ac.cn (J.C.); liuyang6@ime.ac.cn (Y.L.); zhaoyang@ime.ac.cn (Y.Z.); zhanglingqian@ime.ac.cn (L.Z.); maohaiyang@ime.ac.cn (H.M.); 2 University of Chinese Academy of Sciences, Beijing 100049, China; 3 Department of Cellular and Molecular Biology, Beijing Chest Hospital, Capital Medical University, Beijing Tuberculosis and Thoracic Tumor Research Institute, Beijing 101149, China; zhanglina912@126.com

**Keywords:** nanotechnology, circulating tumor cells (CTCs), microfluidic, cell capture, cell release, cell analysis

## Abstract

Circulating tumor cells (CTCs), a type of cancer cell that spreads from primary tumors into human peripheral blood and are considered as a new biomarker of cancer liquid biopsy. It provides the direction for understanding the biology of cancer metastasis and progression. Isolation and analysis of CTCs offer the possibility for early cancer detection and dynamic prognosis monitoring. The extremely low quantity and high heterogeneity of CTCs are the major challenges for the application of CTCs in liquid biopsy. There have been significant research endeavors to develop efficient and reliable approaches to CTC isolation and analysis in the past few decades. With the advancement of microfabrication and nanomaterials, a variety of approaches have now emerged for CTC isolation and analysis on microfluidic platforms combined with nanotechnology. These new approaches show advantages in terms of cell capture efficiency, purity, detection sensitivity and specificity. This review focuses on recent progress in the field of nanotechnology-assisted microfluidics for CTC isolation and detection. Firstly, CTC isolation approaches using nanomaterial-based microfluidic devices are summarized and discussed. The different strategies for CTC release from the devices are specifically outlined. In addition, existing nanotechnology-assisted methods for CTC downstream analysis are summarized. Some perspectives are discussed on the challenges of current methods for CTC studies and promising research directions.

## 1. Introduction

Cancer has become one of the leading causes of death worldwide, and tumor metastasis is the main cause of high cancer mortality [1]. The metastatic process occurs via the transport of malignant tumor cells. Circulating tumor cells (CTCs) are cancer cells that spread through the blood from the primary tumor site [2]. Compared with traditional methods for clinical tumor detection, such as imaging diagnosis, endoscopy and pathological diagnosis, etc., CTC detection has the advantages of noninvasive and dynamic monitoring [3,4]. CTCs are one of the few new tumor molecular markers in cancer diagnosis and therapy assessment and they have been attracting great attention in recent decades. At present, with the expanded understanding of CTCs, their application has moved from the number to the era of molecular typing and cell sequencing [5,6].

The premise of CTC detection is to obtain CTCs from clinical samples. CTCs are extremely rare, with only 1–10 appearing in 1 mL peripheral blood with around 500 million normal blood cells, so isolating and detecting CTCs from the complex and heterogeneous mixtures is a critical task [7]. To date, with the development of micro-electro-mechanical system (MEMS) and micro-total analysis system (μTAS) technologies, various microfluidic platforms featured with chambers, channels and nanostructures have promoted the development of CTC research with the ongoing advances of micro/nanotechnologies. Microfluidic systems have the advantages of small sample volume demands, fast processing times, multiplexing capabilities and large surface-to-volume ratios [8,9,10]. These features offer new opportunities for in vitro cell capture and detection. Hence, it is necessary to perform advanced microfluidic-based approaches to realize the efficient capture and release of rare CTCs for clinical cancer studies and applications.

In recent years, based on the different biophysical and biochemical characteristics of CTCs, the capture methods of CTCs have generally been divided into physical property-based methods (i.e., size, density, adhesion, deformability, dielectric properties, magnetic susceptibility and hydrodynamic properties, etc. [11,12,13,14]) and affinity reaction-based methods (i.e., antibody, aptamer, etc. [15,16]). Many reviews of the different kinds of CTC capture methods have been reported and many platforms have successfully established the detection of CTCs with competitive efficiency and sensitivity [11,15,16,17,18,19,20]. The main advantages of physical property-based capture include the fact that it is label-free, simple and fast. For example, microfilters, inertial microfluidics and deterministic lateral displacement (DLD) [21,22,23,24,25] are typical passive label-free approaches to size-based CTC isolation. There are several limitations of using fluid dynamics methods, mainly due to the low throughput, clogging issues and bulky experimental setup. In addition, acoustophoresis [26], dielectrophoresis [27], magnetophoresis [17] and optical techniques [18] have been used for enhanced active CTC isolation and analysis based on the differences in mechanical properties. Compared to passive methods such as DLD and microfilters, active methods based on the mechanical properties of CTCs have better flexibility and can achieve superior separation resolution. However, such methods lack specificity and are prone to losing tumor cells other than the characteristic parameters.

CTCs also exhibit some unique biochemical properties attributed to the specific tumor markers expressed by CTCs, which can be used to distinguish CTCs from other cells, such as epithelial cell adhesion molecules (EpCAM) and cytokeratin (CK), as well as prostate specific antigen (PSA), epidermal growth factor receptor (EGFR) and carcinoembryonic antigen (CEA), etc. [28,29,30]. Compared with physical property-based methods, immune recognition can specifically isolate cells expressing specific antigens with higher specificity.

Many CTC isolation techniques include two steps, capture and release: the latter has seldom been reviewed and discussed, although it is equally as important as CTC capture for downstream analysis. The isolated CTCs are often adhered to or trapped on the device based on either their biophysical or biochemical properties, as described above. The best way to release these target CTCs from the device still remains a daunting challenge and affects downstream in-depth analysis, such as cell culture or precise manipulation at a single-cell level [31,32,33].

In recent decades, many research and review articles has focused on CTC isolation and analysis using microfluidic devices. A microfluidic device has the features of miniaturization, portability, cost-effectiveness and precise control of the localized microenvironment, which caused it to become one of the mainstream technologies for CTC study [5,8]. These reports either focused on the different methodologies of CTC isolation based on their physical properties [21,22,23,24,25,26,27,34,35,36,37,38,39,40,41,42,43,44,45,46,47,48,49,50,51] or CTC detection using microfluidic devices based on the newly discovered CTC biomarkers and their affinity reaction. Among these studies, nanotechnology-assisted CTC isolation and analysis using microfluidic devices has attracted more and more attention as it combines the advantages of both nanotechnology and microfluidic areas [15,16,33]. Nanostructured-assisted devices for CTC capture provide increased contact area between the cell surface and the nanofeatures of the device, thus allowing for more binding sites and more efficient affinity capture [16]. Under the premise of small-volume fluid handling provided by microfluidic technology, the diversity of nanostructures provides more new targeted strategies for CTC isolation and analysis.

**Figure 1:**
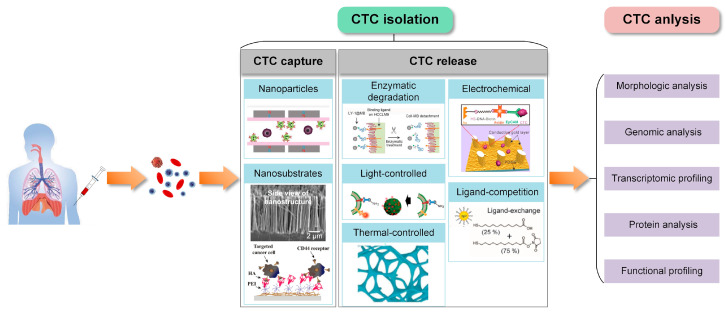
Classification of nanotechnology-assisted methods using microfluidic devices developed for isolation and analysis of circulating tumor cells. All figures are reproduced with permission from [52,53,54,55,56,57,58,59].

In this review, we mainly focus on recent progress in nanotechnology-assisted microfluidic platforms for CTC isolation and analysis (Figure 1). Firstly, we reviewed the development and working principles of typical nanomaterial-based CTC capture techniques. In addition, the performance of each approach in terms of throughput, purity, cell viability and practicality for post-processing are discussed. Secondly, the representative strategies for CTC release are summarized. Lastly, we present application examples of nanotechnology-assisted microfluidic devices which further facilitate downstream analyses of CTCs, especially focusing on the applications of morphologic analysis, gene sequencing, protein analysis and transcriptomic and functional profiling. The major technical progress and application examples that we have reviewed contribute to guiding peers towards many new exciting possibilities in CTC research.

## 2. Nanotechnology-Assisted Circulating Tumor Cell (CTC) Isolation Using Microfluidic Devices

With the in-depth study of the mechanism of tumor metastasis and the continuous improvement of detection technology, a variety of CTC isolation technologies have emerged to utilize the physical and biochemical features of CTCs [16,17,18]. CTC isolation exploits the differences in physical and biochemical properties between CTCs and hematopoietic cells. Microfluidic chip technology provides implementation conditions for integrating multiple operations, including pretreatment, capture, detection, etc., into a single micro device, thus providing a convenient platform for subsequent cell research. Tailoring the nanostructure of the microfluidic device can provide surface areas for affinity ligand immobilization and/or modulate fluidic flow characteristics to enhance the efficiency of CTC capture. For the post-processing analysis, the captured CTCs should remain viable and be released for subsequent analysis. Now, it is important to explore reasonable capture and release platforms under the nanotechnology-assisted microfluidic strategy to realize efficient and high-purity separation and overcome the adhesive forces while keeping cells viable and unperturbed.

### 2.1. Physical and Biochemical Properties of CTCs

The presence of CTCs in cancer patients was first detected in 1869 and their identification was initially performed by a trained cytologist based on chromatin fragmentation and nuclear elongation [34]. Numerous studies in the past decade have shown that CTCs may be used as a marker to predict disease progression and survival in metastatic and possibly even in early-stage cancer patients [2,4]. Despite their great potential, the clinical application of CTCs still faces many hurdles. The isolation of CTCs is particularly challenging due to their extreme rarity, with only a single CTC per billion normal blood cells in blood samples from patients with advanced cancer.

To date, many approaches have been developed for the label-free isolation of target cells utilizing the intrinsic physical properties of CTCs, i.e., size, density, adhesion, deformability, dielectric properties, acoustic properties, magnetic susceptibility and hydrodynamic properties, etc. [11,12,13,14,35,36,37,38,39,40,41,42,43,44]. For the size property, cancer cell lines of solid tumors are much larger than leukocytes [35]. By using the CellSearch™ system, Ligthart et al. also reached the conclusion that leukocytes were two to four times smaller in area than breast, colorectal and prostate cancer cells [36]. However, some studies show a large size overlap between CTCs and leukocytes. Researchers have utilized these features and implemented them in microfluidic devices for CTC isolation. Main different properties of CTCs and normal cells (e.g., leukocytes) are reviewed separately (Table 1).

CTCs express some unique biochemical characteristics, especially for specific biomarkers, which can be used for specific isolation (i.e., cell surface antigen) or detection (i.e., various antigens or mRNAs). Epithelial cell surface markers are the most widely used CTC biomarkers, such as EpCAM (also known as CD326, hea125 and TACSTD1). The specific biomarkers also include more cancer-specific markers, such as HER2neu and MUC1 in breast cancer, androgen receptor (AR), early growth response gene (EGR) and PSA in prostate cancer [45,46,47]. The affinity-based strategies to isolate CTCs depend on the affinity of specific antigens expressed on the cell surface to their corresponding antibodies coating the microfluidic platform’s surface or magnetic substances [28]. The CellSearch system employs a conjugation of EpCAM antibodies to magnetic beads for capturing CTCs through a magnetic field [60]. There is an ongoing discussion about the epithelial–mesenchymal transition (EMT) of tumor cells during dissemination, resulting in a more mesenchymal or even more stem cell-like phenotype [4]. Yu and colleagues proved that EMT exists in human breast cancer specimens, and they used three antibodies (EGFR, HER2 and EpCAM) to capture breast cancer cells more efficiently [48]. Therefore, antibodies against EpCAM cannot detect all tumor subtypes. The major drawback of affinity-based strategies with single marker specificity is that they overlook potentially important CTC subpopulations, thereby jeopardizing the sensitivity of CTC isolation. Immunofluorescence staining is commonly used to differentiate CTCs from the other captured cells to reduce the number of false positives. Typically, CTCs stain positive for EpCAM, CK and DAPI but negative for CD45, although they can stain negative for CK sometimes due to low CK expression. White blood cells stain positive for DAPI and CD45 but negative for EpCAM and CK [28].

The different physical and biochemical properties between CTCs and other cells provide the basis for CTC isolation. To date, various techniques have promoted the isolation and downstream study of CTCs with the ongoing advances of micro/nanotechnologies. There has been interest in using techniques that take advantage of similar scales of micro/nanoscale technologies and intrinsic properties of cells for increased isolation efficiency, purity and cell viability.

**Table 1:** Comparison of physical and biochemical properties between circulating tumor cells (CTCs) and normal cells (e.g., leukocytes).

Properties	CTCs	Normal Cells (e.g., Leukocytes)	Ref.
Size	Average diameter ranging from 15 to 25 μm	Erythrocytes have an average diameter of 7–8.5 μm and leukocytes are 5–13 μm in diameter.	[35,36]
Density	CTCs are mainly deposited in the monocyte enrichment layer.	Erythrocytes and granulocytes have a density > 1.077 g/mL, monocytes and lymphocytes have a density < 1.077 g/mL.	[37,38]
Morphology	Most CTCs from breast, colorectal and prostate cancer have the roundness of 1.5 ± 0.6, 1.5 ± 1.4 and 1.5 ± 0.8, respectively	The roundness of WBCs is 1.8 ± 1.2.	[39]
Deformability	The stiffness ranges from 200 to 2000 Pa.	The stiffness of neutrophils ranges from 156 ± 87 Pa.	[40,41]
Dielectric properties	CTCs have more negative charges.	Different cell types have significant differences in the cell membrane capacitance.	[42,43,44]
Biomarkers	EpCAM, DAPI CK19, HER2, EP, PR and MUC1 (used for breast cancer diagnosis); PSA, AR, EGR and PTEN (used for prostate cancer diagnosis); CK, ASGPR1, N-cadherin, Vimentin, Gpc3, AFP and albumin (used for hepatocellular carcinoma diagnosis)	CD45, DAPI	[45,46,47]

### 2.2. Nanotechnology-Assisted CTC Capture

Over the past few decades, benefiting from the development of nanomaterials and nanotechnology, researchers have designed various nanosubstrate-embedded microchips with the capability of CTC detection and isolation [49]. Cell adhesion significantly affects cell capture yield; it is regulated by surface chemical composition and nanotopography [16,50]. Compared with normal blood cells, tumor cells exhibit a stronger adhesion preference. Nanostructured substrates perform enhanced local topographic interactions that strengthen cell adhesion and large surface areas for grafting capture agents, resulting in improved cell capture efficiency, purity and sensitivity [51,61]. Integrating nanoparticles or nanostructured substrates into microfluidic chips is a promising approach to CTC detection. So far, a variety of nanomaterial-assisted microfluidic chips have been developed for CTC isolation. In this review, we summarize these studies into two types, nanoparticles in suspension and nanostructures on substrates for CTC isolation, which are reviewed below. The performance of typical approaches is summarized in Table 2.

#### 2.2.1. Nanoparticles in Suspensions for CTC Capture

##### Magnetic Nanoparticles

**Figure 2:**
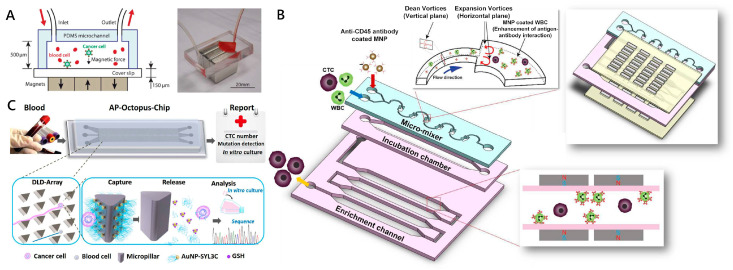
Nanoparticle-embedded microfluidic chips for circulating tumor cell (CTC) capture. (**A**) Schematic showing the operation principle of the microchip for immunomagnetic detection of cancer cells. CTCs in blood were labeled with epithelial cell adhesion molecules (EpCAM) functionalized Fe_3_O_4_ magnetic nanoparticles and captured by the magnetic field as the blood flows through the microchannel. Reproduced with permission from [63]; (**B**) The multivortex micro-mixer inside mixing/magnetic-activated cell sorting (μ-MixMACS) chip enhanced contact frequency between CD45 conjugated magnetic nanoparticles (MNPs) and white blood cells (WBCs). The MACS device captured the MNP-coated WBCs and eluted CTCs through the outlet. Reproduced with permission from [67]; (**C**) Based on the deterministic lateral displacement (DLD) principle, CTCs were forced to cross streamlines and continuously interact with AuNP-aptamer modified micropillar. After capture, the enriched CTCs could be released by the Au-S bond disrupted by excess Glutathione (GSH), which is fully compatible with downstream analysis. Reproduced with permission from [53].

Magnetic nanoparticles (MNPs) are one of the most well-established nanomaterials in immunoaffinity-based CTC detection. Due to the relatively high surface to volume ratio, MNPs functionalized with anti-EpCAM antibody are utilized to bind to target tumor cells for in vitro separation under the external magnetic field [62]. Microfluidic-integrated immunomagnetic assay is a method which can utilize the downscaled immunomagnetic techniques inside the microchannel for efficient separation of cells bound to MNPs, such as Fe_3_O_4_ MNPs [63] and Fe_3_O_4_/Au core/shell MNPs [64]. Zhang’s group developed a microfluidic chip-based CTC capture system in which anti-EpCAM modified Fe_3_O_4_ MNPs’ array inside a microchannel was utilized to identify and separate tumor cells from a whole blood sample (Figure 2A) [63]. This device achieved an average capture rate of 90% for COLO-205 and 86% for SKBR3 cells under an optimal blood flow rate of 10 mL h^−1^, respectively. In order to avoid the effect of red blood cell (RBC) sedimentation on the capture purity, an automated motion-controlled microfluidic system was designed in which magnets were put on top of the microchannel (inverted channel) [65]. Based on a similar mechanism, a series of microfluidic devices and methods for CTC isolation was developed, such as microfluidic magnetic activated cell sorting (μ-MACS) [66], a microfluidic mixing/magnetic-activated cell sorting (μ-MixMACS) device [67] and a wavy-herringbone (wavy-HB)-structured microfluidic device [68]. Lee et al. (Figure 2B) reported a μ-MixMACS device for one-step negative enrichment [67]. On this device, a micromixer could generate multivortexing flows to enhance interaction between CD45 antibody-conjugated MNPs and white blood cells (WBCs), and a magnetic sorter could remove MNPs-coated WBCs. After specifically removing WBCs, more than 85% of the MCF-7 cells were obtained from leukocyte samples. Shi et al. developed a wavy-herringbone (wavy-HB)-structured microfluidic device in which anti-EpCAM coated MNPs were immobilized over the wavy-HB surface under an external magnetic field [68]. A high yield (92%) and a high range of CTC capture efficiency (81–95%) in whole blood samples were obtained using this wavy-HB structured device.

Recent research indicates that CTCs may be heterogeneous when monitoring disease progression. According to the tumor cells with different levels of surface marker expression, Kelley’s group successfully used a microfluidic chip with X-shaped structures to classify CTCs bound to MNPs in whole blood [69,70,71,72], which could effectively capture the subpopulations of anti-EpCAM antibody functionalizing MNP-bound CTCs [72]. By tuning the average flow rate, CTCs with a 100-fold difference in EpCAM expression were captured and transported to spatially distinct zones.

##### Nanoparticles of Gold and Other Materials

Except MNPs, many nanoparticles with various materials, such as Au and SiO_2_ nanoparticles (NPs), were also explored for CTC isolation. Due to their simple synthesis and modification, gold nanoparticles (AuNPs) are generally used for assembling various aptamers to increase the efficiency of CTC capture. Park et al. utilized a thiolated ligand-exchange reaction with AuNPs on a herringbone chip (NP-^HB^ CTC-chip) to efficiently isolate cancer cells from the peripheral blood [52]. The AuNPs were composed of a mixed monolayer of 11-mercaptoundecanoic acid (MUA) and 12-mercaptododecanoic acid N-hydroxysuccinimide ester (NHS). The functionalized AuNPs were immobilized within the herringbone microchannels through reactions with amine groups. The chip with bound NeutrAvidin-NP assemblies was coated with anti-EpCAM via tetravalent biotin-NeutrAvidin binding to facilitate specific tumor cell binding. Compared with a standard ^HB^ CTC-chip, higher capture efficiency (99%) and lower nonspecific binding (35% decrease) were observed for PC-3 cells in the NP-^HB^ CTC-chip. Song et al. (Figure 2C) engineered a DLD-patterned microfluidic chip modified with interfaces mimicking the multivalent tentacles of an octopus using aptamer-functionalized AuNPs [53]. Compared with a monovalent aptamer modified chip, the capture efficiency of the device was enhanced by more than three times under the synergistic interaction between the AuNP aptamer and EpCAM. Furthermore, the AuNP aptamer interfaces can achieve 80% efficiency and 96% viability of tumor cells via a thiol exchange reaction, which was fully compatible with downstream mutation detection and CTC culture. Based on the method of affinity separation, Zhou et al. employed a polydimethylsiloxane (PDMS)-based HB chip with SiO_2_ NPs for cancer cell isolation and purification [73]. Anti-CD71 was used as the capture ligand in the HB chip to detect a wide range of cancer cell lines. Compared with the normal HB chip, the capture efficiency of the NP-modified HB chip exhibited an average increase of 16%. Moreover, the capture purity of the chip was also elevated at all spike percentages ranging from 1% to 20%. In general, both magnetic and non-magnetic nano-immune enrichment methods take advantage of the larger surface area of NPs, increasing contact frequency between target cells and corresponding antibodies.

#### 2.2.2. Nanostructures on Substrates for CTC Capture

##### Nanopillars, Nanowires, Nanofibers

**Figure 3:**
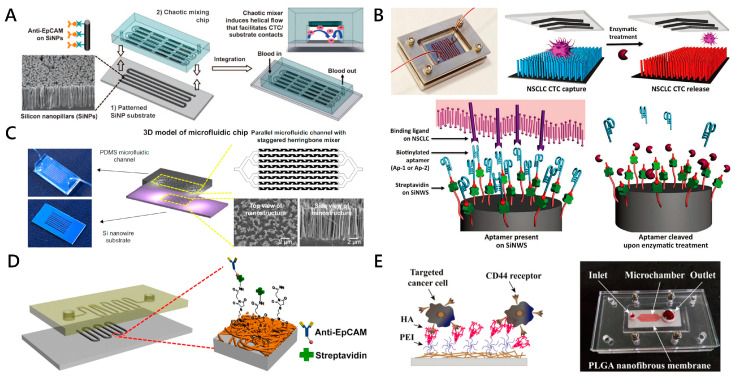
Nanopillars, nanowires and nanofiber-embedded microfluidic chips for CTC capture. (**A**) A CTC capture platform consisting of a polydimethylsiloxane (PDMS) chaotic micromixer covered and a patterned SiNP-embedded substrate with an anti-EpCAM coating. Reproduced with permission from [77]; (**B**) “NanoVelcro” Chip integrating an aptamer-coated SiNW substrate and an overlaid chaotic mixer for capturing and releasing non-small cell lung cancer patients (NSCLC) CTCs from blood samples. Reproduced with permission from [78]; (**C**) Unique staggered herringbone structure and Pe-SiNWs were adopted in microfluidic device to enhance the interactions between cells and channel substrate. Reproduced with permission from [54]; (**D**) A microchip comprised of a serpentine PDMS microchannel and a glass substrate with MnO_2_ nanofibers to capture anti-EpCAM positive-expressing cells. Reproduced with permission from [81]; (**E**) The microfluidic device embedding functionalized poly(lactic-co-glycolic acid) (PLGA) nanofibers for capturing CD44 receptor-overexpressing cancer cells. The functionalized PLGA nanofibrous membrane and hexagon-shaped microfluidic chamber were tightened by screws in between two self-machined polymethyl methacrylate (PMMA) plates. Reproduced with permission from [55].

Except the abovementioned nanoparticle in suspension to enhance the CTC isolation efficiency, there are also numerous nanostructures on substrates which were studied for CTC capture. An important characteristic of PDMS microfluidic devices is that they enable easy integration with a variety of nanostructured substrates functionalized for CTC capture [74]. Numerous studies have shown that substrates with high-aspect-ratio nanostructures (a ratio of height to diameter ≥ 2:1), including nanopillars, nanowires and nanofibers, have an important influence on cell adhesion, migration, differentiation and other behaviors [75,76]. In the presence of specific antibodies against the target cell, the nanopillar, nanowire and nanofiber-based biological interfaces increase the surface contact with cell-surface components (filopodia or lamellipodia), allowing more binding sites to achieve highly efficient affinity-based CTC capture [51]. In this field, Wang et al. (Figure 3A) presented a microfluidic chip with an 88 cm long serpentine chaotic mixing channel, which was integrated with anti-EpCAM coated silicon nanopillars (SiNPs) [77]. This chaotic micromixer could induce vertical flow, which significantly facilitated the contact frequency between CTCs and the SiNPs’ substrate. A capture yield of more than 95% of the MCF-7 cell line (an EpCAM-positive breast cancer cell line) from artificial blood samples was achieved at an optimal flow rate of 1.0 mL h^−1^. Shen et al. (Figure 3B) reported a novel microfluidic chip for CTC capture. The device, which was named “NanoVelcro” chip, was composed of a patterned SiNW substrate coated with CTC selective DNA aptamer and a chaotic mixer with a 22 cm long microchannel [78]. The chip achieved above 80% capture efficiency for A549 cells from artificial blood samples, at a flow rate of 1.0 mL h^−1^. For the captured A549 cells, cell viability was 78–83%. They also integrated multiple strategies and technologies of unique staggered herringbone mixer, peptide-silicon nanowires’ substrate (Pe-SiNWs) and enzymatic release into a microfluidic chip (Figure 3C) [54]. When the flow rate was 1.0 mL h^−1^, the Pe-SiNWs modified by EpCAM and specific peptides CKAAKN demonstrated the highest capture efficiency of 91.4%. More importantly, the device retained 82.6% capture efficiency even when the flow rate reached 5.0 mL h^−1^. The CTC detection system demonstrated the merits of high throughput and high sensitivity. On the basis of previous experience using the “NanoVelcro” substrate, Dong et al. designed a covalent chemistry-based SiNW substrate (“Click Chip”) to address the unmet clinical need of quantifying gene rearrangements in CTCs [79].

Unlike nanopillars and nanowires, nanofibers with extremely high aspect ratios are deposited horizontally on the substrates [80]. The combinatory strategy using nanofibers and microfluidic technology improves the efficiency and specificity of CTC detection, which is better than using nanofibers alone. Liu et al. (Figure 3D) designed a microchip based on the etchable MnO_2_ nanofibers that are integrated into microchannels [81]. The electrospinning MnO_2_ nanofiber net mediated by anti-EpCAM can provide an extra cellular matrix (ECM) mimicking the topographical environment for high capture ability. At a flow rate of 0.1 mL h^−1^, the microchip with 20 nm diameter of nanofibers can achieve CTC affinity capture with efficiency of 80%. In order to effectively capture CTCs of different origins from blood samples, Xu’s group (Figure 3E) fabricated a thin sheet of poly(lactic-co-glycolic acid) (PLGA) nanofibers which were embedded in a microfluidic chip [55]. The functional electrospun PLGA nanofibers modified with hyaluronic acid (HA) through polyethyleneimine (PEI) linkers exhibited specificity to cancer cells overexpressing CD44 receptors via ligand–receptor interactions. The results showed that the capture efficiency of HeLa, A549 and MCF-7 cells could reach more than 80% with a 1.0 mL h^−1^ flow rate. The HeLa cells captured on the nanofibers could maintain viability in a dynamic culture microenvironment after four days of culture.

**Table 2:** Comparison of typical nanomaterial-based CTC isolation approaches.

Microchip-Based Nanosubstrate	Materials	Optimal Throughput	Capture Efficiency %	Purity %	Viability %	Cancer Cell Type	Highlights	Ref.
Magnetic nanoparticles	Fe_3_O_4_ MNPs + anti-EpCAM antibody	10 mL/h	90% (COLO-205 cells) 86% (SKBR3 cells)	N/A	N/A	COLO-205 and SKBR3 cells	Effective capture achieved by arrayed magnets with alternate polarities	[63]
		540 μL/h	80~90%	≈91%	>95%	HCT-116 cells	Wavy-herringbone structure for effectively and selectively capturing and releasing CTCs	[68]
Gold and other materials nanoparticles	Au NPs + Aptamer-SYL3C	1 mL/h	74~84%	99.99% (WBC removal rate)	96% (SW480 cells)	SW480, LNCap and KATO III cells	Multivalent aptamer–antigen binding; rotated triangular micropillars based on the DLD principle	[53]
	SiO_2_ NPs + CD71 antibody	N/A	94 ± 3% (HL-60 cells) 86 ± 3% (Ramos cells) 89 ± 9% (Jurkat cells) 81 ± 6% (MDA-231 cells) 74 ± 14% (PC-3 cells)	41~65% (COG-LL-332 cells)	N/A	HL-60, Ramos, Jurkat, MDA-231, PC-3 cells	High capture purity; a wide range of cell separations	[73]
Nanopillars, Nanowires	SiNPs + anti-EpCAM antibody	1 mL/h	95%	N/A	N/A	MCF-7 cells	High efficiency achieved with embedded chevron-shaped micropatterns	[77]
	SiNWs + CKAAKN peptides and anti-EpCAM antibody	1 mL/h	≈96%	≈26%	≈94%	BxPC3, Panc-1 and AsPC-1 cells	Novel biological method of enzymatic release	[54]
	SiNWs + anti-EpCAM antibody	1 mL/h	88~94%	≈200 WBCs	N/A	NSCLC cells	Click reaction; Disulfide cleavage-mediated CTC capture	[79]
Nanofibers	MnO_2_ + anti-EpCAM antibody	0.1 mL/h	80%	N/A	≈90%	MCF-7 cells	CTC release by oxalic acid dissolution of MnO_2_	[81]
	PLGA + Hyaluronic acid	1 mL/h	>80%	N/A	N/A	HeLa, A549 and MCF-7 cells	Specific capture of CD44-positive tumor cells	[55]
Other nanoroughened structures	GO nanosheets + anti-EpCAM antibody	1 mL/h	73%	N/A	N/A	PC-3 cells	Higher sensitivity; Gentle capture	[82,83]
	ZnO/Al_2_O_3_ HS-MSA + anti-EpCAM antibody	N/A	≈85%	≈2.6% (per mL artificial CTCs blood samples)	>95%	MCF-7 cells	3D micro/nanostructures; in situ extracellular drug delivery of the captured CTCs	[84,85]
	ZnO nanograss + anti-EpCAM antibody	N/A	>80%	N/A	90%~95%	MCF-7 and MDA-MB231 cells	Self-sterilizing and regeneratable	[86]

##### Other Nanoroughened Structures

**Figure 4:**
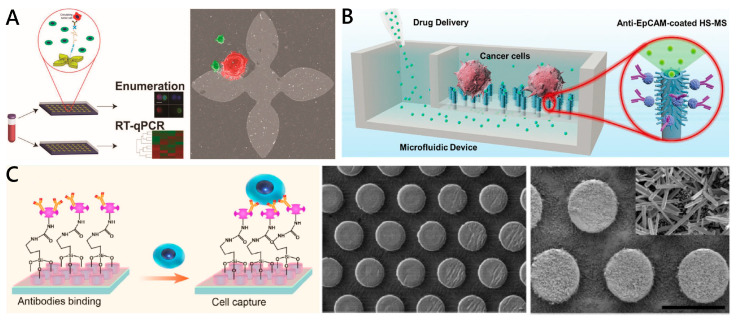
Other nanoroughened structure-embedded microchips for CTC detection. (**A**) Microfluidic graphene oxide-based device (GO chip) enables isolation of prostate CTCs. Captured CTCs and extracted RNA from parallel GO chips were utilized to determine CTC characteristics associated with progression and survival in advanced prostate cancer. Reproduced with Wiley OA’s permission from [82]; (**B**) The anti-EpCAM coated hierarchical spiky microstraw arrays (HS-MSA) were integrated into the microfluidic device. The device has the capabilities of capturing cancer cells and in situ chemically manipulating the captured cells. Reproduced with permission from [84]; (**C**) The ivy-like hierarchical roughened ZnO nanograss substrate was directly integrated into the microfluidic device and enables effective CTC capture during incubation in a mildly acidic solution. Reproduced with permission from [86].

As a new type of 2D nanomaterial, graphene oxide (GO) shows very interesting characteristics for biomedical, nanomedicine and tumor treatment. Kozminsky et al. (Figure 4A) designed a GO-based chip (GO Chip) to separate CTCs and CTC clusters from the whole blood samples of prostate cancer patients [82,83]. The parallel GO chips respectively utilized captured CTCs and extracted RNA to achieve CTC enumeration and RNA expression in a clinical cohort using an extensive 96-gene panel. Hierarchical nanostructures generally are composed of either nanoscale building blocks in multiple dimensions or multiscale. Due to the advantages of high surface contact areas and synergistic interactions (at both nanoscale and microscale), hierarchical nanostructure-embedded substrates play an important role in capturing CTCs. Xie’s group (Figure 4B) fabricated a hierarchical spiky microstraw array (HS-MSA)-integrated microfluidic device [84]. After modification with anti-EpCAM, the hierarchical structure of HS-MSA possessed the capabilities of cancer cell capture and in situ chemical manipulation of the captured cells. The device achieved high cell capture efficiency (≈84%) and extracellular drug delivery of staurosporine (STS) to the captured CTCs. Motivated by the versatility of nanostraws, they also developed a multifunctional branched nanostraw-electroporation platform that could regulate and monitor their intracellular activities in a real-time and in situ manner [85]. Due to the high antibacterial effect of the zinc oxide (ZnO) material, Hui et al. developed a microfluidic chip that integrated with an ivy-like hierarchical roughened ZnO nanograss for CTC capture and release (Figure 4C) [86]. Mean capture yields of MCF-7 and MDA-MB231 cells were all greater than 80% in both phosphate buffer saline (PBS) and processed blood. Approximately 90–95% of the captured cells were viable and recovered during the release process. Therefore, the multifunctional CTC detection platform has clinical significance for personalized cancer therapy.

### 2.3. Approaches to CTC Release from Nanostructures

At present, most of the analysis of captured cells is done by directly lysing the cells on the surface of the device for downstream enzymatic reactions to measure DNA, mRNA, miRNA, ncRNA and proteins. However, the value of captured rare cells is much more than that. CTC downstream analysis has gained increasing attention and become the priority of CTC studies. One of the important premises of CTC downstream analysis is to release the captured CTCs from trapping structures or affinity-adhered substrates while keeping cells viable and unperturbed [32]. For cell-affinity based methods, releasing CTCs from the captured site is still challenging. In recent years, great efforts have been devoted to developing different release methods based on corresponding CTC capture principles. In this section, we focus on the different approaches to CTC release from nanotechnology-assisted microfluidic devices. Also, the typical CTC release approaches are reviewed in Table 3.

#### 2.3.1. Flowing Fluid-Mediated CTC Release

The shear stress generated by the fluid is widely used to separate attached cells. When fluid force can overcome cell adhesion, cells are released from the surface of the microfluidic fluid flow. Flowing fluid-mediated CTC release is easy to achieve, and a well-sealed microfluidic device and a programmable syringe pump is usually sufficient. Therefore, this approach has the main advantages of simplicity and inexpensive nature of pressurized flow. However, many factors still need to be considered in experimental practice, such as antibody type, receptor density on the cell membrane and substrate geometry, in order to obtain the appropriate cell shear force. It is impractical to precisely calculate the shear stress which would be required to release the captured cells. The effects of flow rate, flow acceleration and flow type on cell detachment have been studied before [12,87,88] (Abu-Reesh and Kargi, 1989; Cheung et al. 2009; Lu et al. 2004). Keon et al. tested the function of the fraction of adherent cancer cells (MCF-7 and MCF-10A) on the nanosubstrate and flow rate in order to evaluate the relationship between shear force and cell adhesion [89]. Shear stress has different thresholds for different cells. Normally, cells attached to the device surface require a relatively high detachment force, and shear stress also will affect cell activity and interfere with the microenvironment of cells [89]. The morphology of cells will change under shear stress, such as an increase in cell membrane tension and surface area. It has also been found that shear stress can induce cell differentiation and promote cell proliferation, adhesion and migration [88].

#### 2.3.2. Enzymatic Degradation-Based CTC Release

**Figure 5:**
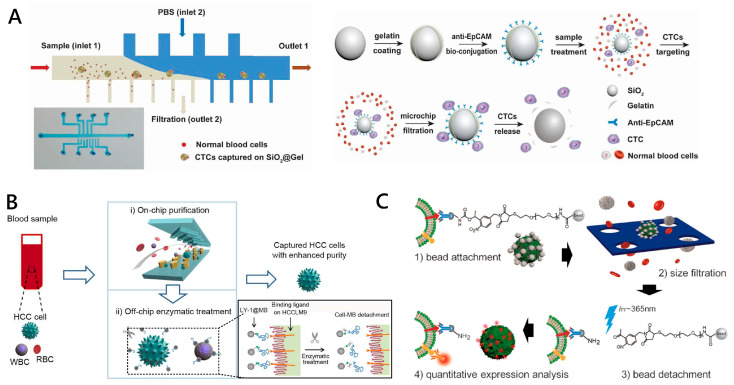
Working principles of CTC release via (**A**), (**B**) enzymatic degradation and (**C**) light-controlling strategy. (**A**) The microchip combined the hydrodynamic drag force and silica microbeads coated with gelatin and anti-EpCAM to realize the isolation of CTCs from other normal blood cells. The release of attached CTCs is realized by the degradation of gelatin by matrix MMP-9 enzyme. Reproduced with permission from [95]; (**B**) The release of CTCs is realized by the exonuclease treatment of the LY-1 aptamer-conjugated microbeads. Reproduced with permission from [56]; (**C**) The platform for CTC isolation, release and in situ analysis combined the high pore density filter and magnetic beads modified with photosensitive molecules. Reproduced with permission from [57].

In this area, trypsinization of cell membrane protein is the main strategy to release CTCs. Cells are resuspended by exposure to 0.25% trypsin in 0.02% ethylene diamine tetraacetic acid (EDTA) at 37 °C for a few minutes, followed by trypsin deactivation [90]. However, long trypsinization can degrade cell surface markers and damage the membrane structure, so it is not conducive to subsequent cell analysis. Degradable biopolymer is also used as the linker of CTC capture, which can be digested by the corresponding enzyme to release CTC from the capture matrix without affecting cell composition, structure and microenvironment. Gelatin and collagen sequences as natural proteins extracted from animal collagen have excellent biocompatibility and biodegradation ability. They can be degraded by matrix metalloproteinases-9 (MMP-9) [91,92,93,94,95]. For example, a biofunctional sacrificial hydrogel-coated microfluidic chip was reported [93]. The patterned hydrogel can be dissolved via backbone cleavage with alginate lyase. The device enables the highly efficient release of isolated cells (99% ± 1%) with high cell viability (98.9% ± 0.3%). Zhao’s group applied SiO_2_@Gel microbeads integrated into a microfluidic filter to isolate CTCs from normal blood cells (Figure 5A). The gelatin-coated silica microbeads (SiO_2_@Gel MBs) of 50 μm in size conjugated to CTC-specific antibodies (anti-EpCAM) and were then used to target EpCAM-positive CTCs via the significant difference in the size of normal blood cells. The gelatin coated on the surface of MBs was degraded by matrix (MMP-9) enzyme to realize the captured CTC with high purity [56].

As a biological recognition molecule, aptamers have many advantages in the capture and identification of CTCs [89]. More importantly, chemically unstabilized aptamers can easily change their conformation and lose their affinity and specificity so as to release the captured CTCs. Nuclease digestion can be applied to cleave nucleic acid chains by breaking phosphodiesters when aptamers are used as the linkers of CTC capture. The addition of restriction exonuclease and endonuclease can cut off the connection between the capture reagent and substrate. For example, Hao and co-workers applied aptamer-modified magnetic beads (MBs) combined with a magnetic-controlled microfluidic chip to isolate hepatocellular carcinoma CTCs [56]. The release strategy can be divided into two steps. In the first step, the captured cells were simply released from the Ni micropillar substrate by removing the magnetic field and flowing fluid in the channels. The initial release efficiency is around 93%. In the second step, the collected tumor cells were released from the attached MBs after exonuclease treatment of 30 min. The final recovery rate reached ~68% and purity reached ~61% (Figure 5B). It is worth mentioning that nuclease will have an impact on cell viability, so precise control of nuclease concentrations and reaction time to balance the release efficiency and cell viability are needed.

#### 2.3.3. Light-Controlled CTC Release

By connecting a photocleavable linker (PC-linker) to the substrate and the capture material, it is possible to release the CTC by light. The light-sensitive molecules of the PC-linker are broken under ultraviolet or near-infrared radiation and cells detached from the light-responsive polymer surface. These light-response-based approaches are widely used in cell release research due to the advantages of precise control and low invasion. For the adjustment of irradiation parameters, such as irradiation intensity, irradiation wavelength, irradiation time, etc., which can provide precise control of cell release. Lee et al. modified the antibody by attaching an o-nitrobenzyl group as a PC-linker to the surface of microparticles and achieved 98.4% release efficiency and 97% viability of cancer cells by separation based on the physical size of the microparticles [57] (Figure 5C). Photosensitive molecules from the nitrobenzyl group can cause photolysis reaction to both ultraviolet and near-infrared light. Another coumarin group has more efficient two-photon absorption and stronger penetrating power to near-infrared, which is more friendly to cells. Lu et al. selected 7-aminocoumarin as the PC-linker to prepare a light-responsive immunomagnetic nanocarrier for capturing and releasing CTCs [96]. Under ultraviolet and near-infrared (NIR) irradiation, approximately 73% and 52% of the captured cells are released, and the cell viability is 90% and 97%, respectively. Currently, exploring the realization of NIR light-responsive molecules with low cell damage CTC release is a major trend. Light-controlled release provides a new and effective method under photosensitive surface and light irradiation. However, the requirements of safety storage of photosensitive materials, expensive equipment and experienced experimental skills may limit its application to a small automated analysis device for point of care.

#### 2.3.4. Thermal-Controlled CTC Release

**Figure 6:**
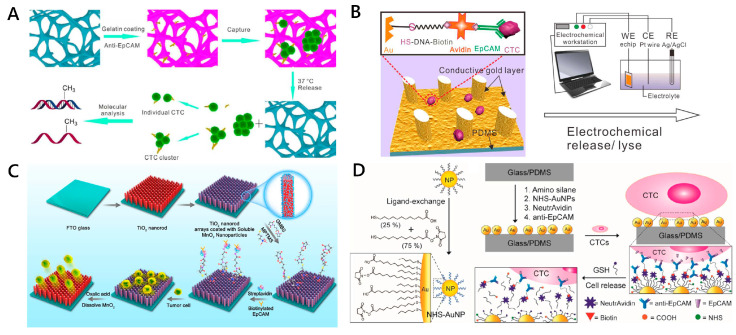
Working principles of CTC release via (**A**) thermal-controlling; (**B**) electrochemical, (**C**) chemical reagent-triggering and (**D**) ligand competition strategy. (**A**) Gelatin-coated 3D PDMS scaffold chip for capture and release of individual and cluster CTCs. Gelatin dissolves at physiological temperature (37 °C), allowing the cell-friendly release of CTCs for further analysis. Reproduced with permission from [58]; (**B**) Electrochemistry microchip with gold layer-coated micropillar array for CTC capture and release. Reproduced with permission from [59]; (**C**) TiO_2_ nanorod arrays coated with soluble MnO2 nanoparticles for CTC release. Reproduced with permission from [104]; (**D**) Thiolated ligand-competitive release system using GSH. Reproduced with permission from [52].

Similar to light-controlled CTC release, thermal-controlled cell capture, reversible cell adhesion and release can be achieved on the thermosensitive substrate through temperature control. Poly(N-isopropyacrylamide) (PIPAAm) is a widely used thermo-responsive polymer which can achieve reversible surface hydrophobic-to-hydrophilic switch at a lower critical solution temperature (LCST) (e.g., 32 °C) [97]. Liu et al. coated a layer of PNIPAAm on silicon nanopillars and modified the antibody on PNIPAAm by the anchor biotin-BSA to isolate MCF-7 cells [98]. The unique thermoresponsive PNIPAAm-modified nanopillars realized reversible cell capture and release by alternating temperatures between 37 °C and 20 °C. It was reported that 98.8% of captured MCF-7 cells were released from the nanostructured surfaces without cell damage. Gelatin is another thermally responsive material that can be used in CTC release. It undergoes a transition from gel to soliquid at an LCST of around 33 °C [58,99]. Chen and his colleagues developed a 3D scaffold chip with gelatin coating for capturing and releasing individual CTCs and CTC clusters [58]. Thermosensitive gelatin hydrogel was uniformly coated on the scaffold, which, modified with the anti-EpCAM antibody, dissolves at 37 °C quickly, and the captured cells are gently released from the chip, with high viability (>90%) and 86% release efficiency (Figure 6A). In general, strategies based on thermally responsive polymers can release CTCs under mild temperature stimuli, providing almost complete CTCs for downstream analysis [33].

The affinity binding between aptamers and biomolecules can be strongly temperature-dependent, allowing for thermally controlled enrichment and release of biomolecules. The specific interaction between aptamers and cells is also usually affected by temperature, which allows thermal-controlled enrichment and release of cells. For example, Zhu et al. developed an aptamer-functionalized microfluidic chip for the isolation and subsequent thermal-controlled release of tumor cells [100]. The aptamer-grafted PDMS layer can release around 80% of the captured cells at 48 °C after rinsing with PBS. When the temperature drops from 60 °C to room temperature, the aptamer will gradually form the initial conformation, which can be used for cell capture again. Some aptamer-based platforms need to reach high temperatures to capture and release cells and this condition will significantly affect the viability of cells that make practical release difficult. For the thermal-controlled CTC release, the simply changing operation and easy to control conditions make it possible for CTC release. However, additional temperature-controlled equipment is necessary. Moreover, it is necessary to select suitable thermosensitive molecules according to the temperature tolerance of specific cells, in order to reduce the impact on their biological characteristics due to changes in the viability of the cells.

#### 2.3.5. Electrochemical and Chemical Reagent-Triggered CTC Release

Electrochemical approaches have been widely used in CTC release, with high efficiency and rapid reaction. This kind of approach contains two essential parts: self-assembled monolayers (SAM) and a conductive metal substrate [95]. Thiol-containing ligand-assembled gold surfaces have been used to pattern cells and control cell separation because of the strong bonding and reducible cleavage of Au-S bonds [59,101]. Liu and co-workers designed a cactus-like PDMS gold-coated electrochemical microchip (echip) which used thiol DNA-biotin as a linker to modify EpCAM-specific monoclonal antibodies [59]. The device achieved capture efficiency of over 93%. The rapid release of the captured tumor cells was achieved by reducing the thiol-gold bond at −1.2 V conditions. The viability of the release cells was up to 95%, enabling reculture and proliferation (Figure 6B). Recently, conductive polymers, such as polypyrrole or poly(3,4-ethylenedioxythiophene) (PEDOT), have been most widely used in bioelectronic interfaces due to their excellent electrical transport properties, biocompatibility and manufacturing flexibility [102,103]. Hsiao et al. proposed a 3D conducting polymer bioelectronic interface based on the electrochemical doping/dedoping properties of PEDOT to realize isolation and electrically triggered release of CTCs. The high-aspect-ratio PEDOT-based nanorod arrays fabricated on indium tin oxide (ITO) electrodes were integrated the biotinylated poly-(L)-lysine-graft-poly-ethylene-glycol (PLL-g-PEG-biotin) coating for the capture and release of CTCs. More than 90% of the captured cells were released with 20 cycles of cyclic voltammetry, sweeping from −0.8 to 0.5 V, while maintaining high cell viability [103]. These strategies provide efficient CTC release and a time-saving process. However, the disadvantages of electrochemical release strategies are the elaborated fabrication of the conducting substrate and complex modification of capture ligands, which make the practical operation difficult. Meanwhile, the external electrical stimulation may have an effect on the cell.

Chemical reagent-triggered degradable nanosubstrates provide a new direction for CTC release [82,104,105]. Li et al. fabricated a CTC capture platform based on TiO_2_ nanorod arrays coated with transparent MnO_2_ nanoparticles [104]. The modified MnO_2_ nanoparticles on the substrate were etched with oxalic acid at a low concentration at room temperature; thus, the attached cells could be released. The platform achieved isolation efficiency of 92.9%, release efficiency of 89.9%, and the viability of released cancer cells exceeded 90% (Figure 6C). Similarly, a ZnO-based substrate with chemical degradability can be dissolved under 1% sodium citrate (pH 6.5); thus, it can be applied to cell release [86]. These approaches apply acids to dissolve nanosubstrates; the concentration of acid needs to be elaborately optimized to avoid cell damage.

#### 2.3.6. Ligand Competition for CTC Release

The mutual competition of molecules can destroy the originally stable cell capture system, thereby achieving cell release. Alginate aqueous solutions can form hydrogels with the aid of Ca^2+^, while alginate hydrogel can be dissolved with the aid of EDTA. Therefore Ca^2+^ and EDTA can be used for reversible ligand competition for CTC release. Xie et al. realized EDTA-assisted cell release based on Ca^2+^-alginate chemistry [106]. The alginate coating was deposited on the surface of fluorescent magnetic nanospheres, and anti-EpCAM was connected to the surface of nanospheres by the presence of Ca^2+^. After magnetic separation, the captured tumor cells were released after being treated with 50 × 10^−3^ M EDTA for 5 min, with release efficiency of ≈65% and viability of ≈70%. However, EDTA could affect the functions of cellular surface adhesion molecules, so it was necessary to choose more stable competitive reagents.

In the presence of excess thiol molecules, the interactions of meta–thiol can be easily disrupted. Based on this principle, Hammond and co-workers utilize a thiolated ligand-competitive reaction with modified gold nanoparticles (AuNPs) on a herringbone chip (NP-HBCTC-chip) to isolate and release cancer cells from whole blood [65]. Glutathione (GSH) and excess thiol molecules were utilized to competitively exchange original thiol ligands, resulting in CTC release with over 90% efficiency and 78% cell viability (Figure 6D). Shen et al. reported a phenylboronic acid (PBA)-grafted PEDOT NanoVelcro chip with anti-EpCAM antibody conjugated onto the nanosubstrate of the chip via PBA–oligosaccharide binding [107]. The introduction of glycan with stronger affinity to PBA results in competitive binding, allowing effective CTC release. The ligand competition release strategy has various candidate approaches, allowing the selection of an appropriate approach according to the application demands and available experimental conditions. In addition, reversible capture and release of CTCs can be realized under this strategy. However, some competing molecules may affect the functions of cellular adhesion molecules.

**Table 3:** Comparison of typical CTC release approaches under nanotechnology-assisted microfluidic devices.

Mechanism	Material	Reference	Treatment Conditions	Capture Efficiency	Release Efficiency	Cell Viability	Cell Types
Enzymatic Degradation	SiO_2_@Gelbead + PDMS microfluidic filter	[95]	Solution of MMP-9 enzyme	75%	97.2% ± 2.1%	>97%	HCT116 cells
	PDMS herringbone structure + aptamer-conjugated MBs were trapped on the substrate of Ni pillar arrays	[56]	Exonuclease at 37 °C for 15 min	~91%	~68.4%	NA	HCCLM9 cells
Light-Controlled	Detachable beads conjugated to antiEpCAM antibodies + 8 μm microporemembrane	[57]	Irradiation from an LED light source (λ = 365 nm)	89.1%	98.4%	97%	SKBR3 cells
	7-Aminocoumarin reacted with biotin to bridge the antiEpCAM antibogshdy and streptavidin (SA) modified MBs	[96]	UV or near-infrared (NIR) light irradiation	~91%	73% ± 4% and 52% ± 6%	90% and 97%	HeLa, MCF-7 and SKBR3 cells
Thermal-Controlled	Silicon nanopillars coated with PNIPAAm + antibody	[98]	Alternating temperatures between 37 °C and 20 °C	97.3% ± 0.4%	98.8% ± 0.5%	~95%	MCF-7 cells
	3D PDMS scaffold chip with gelatin coating + antibody	[58]	Incubated for 10 min at 37 °C	88%	86%	90%	MCF-7 cells and Hela cells
	Aptamer-functionalized tapered chamber + temperature control chip	[100]	Heated at 48 °C for 2 min	~92%	~80%	Normal proliferation	CCRF-CEM cells
Electrochemical and Chemical Reagent-Triggered	Thiol-containing ligand assembled gold surfaces of PDMS micropillar-array	[59]	At voltage of −1.2 V for 10 min	85~100%	>93%	>95%	MCF-7 cells and HepG2 cells
	3D PEDOT-based nanorod arrays coated with PLL-g-PEG-biotin	[102]	20 cycles of cyclic voltammetry sweeping from −0.8 to 0.5 V	NA	>90%	~90%	MCF-7 cells and Hela cells
	TiO_2_ nanorod arrays coated with transparent MnO_2_ nanoparticles	[104]	Oxalic acid solution	>60%	89.9%	>90%	SW480 and MCF-7 cells
	Anti-EpCAM functionalized zinc oxide nanowires on glass substrate	[105]	1% sodium citrate in water (pH 6.5)	90% ± 1%	88% ± 4%	92% ± 1%	MCF-7 cell
Ligand Competition	Anti-EpCAM- deposit alginate coating on fluorescent-magnetic nanospheres	[106]	EDTA treatment	>85%	~65%	~70%	SKBR3 cells
	Thiol-modified AuNPs + anti-EpCAM assembled on a PDMS HB chip	[65]	GSH	Average of 68% ± 29.2% (MDA-MB-231) and 72% ± 26.4% (PC3 cells)	92% (PC3) and 91% (MDA-MB-231)	78% (MDA-MB-231) and 87% (PC3)	PC3 cells and MDA-MB-231 cells
	PBA-grafted PEDOT NanoVelcro chip	[107]	0.5 M sorbitol solution	~70%	>95%	>96%	LNCaP and PC3 cells

## 3. Nanotechnology-Assisted CTC Analysis in Microfluidic Chips

The major advantage of “liquid biopsy” using CTCs as a biomarker is the ability to evaluate tumor characteristics in a non-invasive and highly feasible way, paving the way for early screening of cancer and personalized oncology instead of biopsy [16]. Downstream molecular analysis and cell function profiling of isolated CTCs can reveal the biological mechanism of tumorigenesis, tumor progression and metastasis, analyze cell heterogeneity, evaluate therapeutic efficacy, assess drug resistance, analyze prognosis, etc. Nanotechnology-assisted microfluidic platforms possess higher sensitivity for CTC isolation and better compatibility with downstream analysis. They have potential application value in clinical tumor exploration. In this section, we highlight several applications of nanostructure-assisted microfluidic platforms for CTC analysis.

### 3.1. CTC Morphologic Analysis

**Figure 7:**
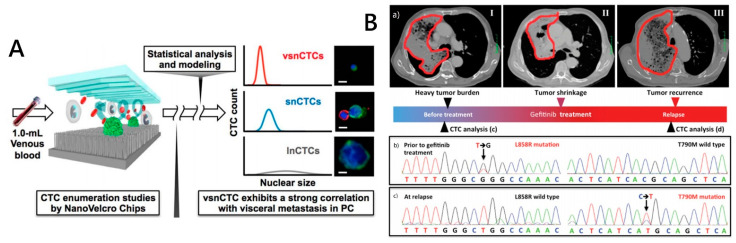
Morphologic analysis (**A**) and genomic analysis (**B**) of CTCs isolated by NanoVelcro chips. (**A**) Three subpopulations of CTCs were identified by morphological analysis of CTCs captured by NanoVelcro chip. Reproduced with permission from [109]; (**B**) Monitoring of computed tomography image and hotspot mutations in epidermal growth factor receptor (EGFR) gene of a NSCLC patient during the three stages of before, after drug treatment and drug resistance. Reproduced with permission from [114].

Many nanostructured substrate-embedded devices have small vertical variation and large contact surface areas of the isolated CTCs, which provide effective imaging conditions for downstream analysis. With the aid of high-resolution imaging technologies, morphologic variations of CTCs and distinct phenotypic subpopulations can be identified, many of which may be closely related to tumor metastasis. Recently, there has been increasing interest in developing computational methods to assist in the morphologic categorization and identification of microscopic images of single cells [108]. Chen et al. perform detailed morphologic analysis for cellular and subcellular features of captured prostate cancer CTCs under the NanoVelcro chip (with anti-EpCAM conjugation) [109]. The principles of CTC capture and release of the NanoVelcro chip are described in Section 2.2.2 and Section 2.3.6. The mathematical modeling and unsupervised clustering on the CTC nuclear size distribution identified three distinct subpopulations of CTCs: large nuclear CTCs (lnCTCs), small nuclear CTCs (snCTCs) and very small nuclear CTCs (vsnCTCs) (Figure 7A). Serial enumeration studies suggested that the emergence of vsnCTCs correlates with the presence of visceral metastases and should be explored as a biomarker of prostate cancer. Eric M. et al. proposed a machine-learning approach called “in silico labeling” (ISL) which reliably predicts fluorescent labels (e.g., DAPI, Hoechst) from transmitted light images of unlabeled fixed cells [110]. ISL can also predict a range of labels, such as nuclei, cell type and cell state, and enable longitudinal fluorescence-like imaging with no additional sample preparation and minimal impact on cells. This deep learning system provides a new perspective for morphological analysis of captured CTCs on nanosubstrates.

### 3.2. CTC Genomic Analysis

Genomic analysis of CTCs advances our understanding of the molecular mechanisms underlying tumor metastasis, relapse and drug resistance and helps us to develop new targeted anticancer therapies [5,33]. Conventional preprocessing techniques for CTC sequencing include limited dilution, micromanipulation, fluorescence-activated cell flow cytometry, etc. The combination of microfluidic and nanotechnology can provide some new strategies for the pretreatment of CTC genomic analysis, which makes the acquisition of CTCs easier and can be combined with many analysis methods. In particular, some nanotechnology-assisted platforms coupled with cell release strategies have specifically obtained the target CTCs for target-specific polymerase chain reaction (PCR) and/or whole genome amplification (WGA) and have been further subjected to mutation analysis.

For oncogenic driver mutation detection, researchers often focus on the hotspot mutations in BRAF [111], KRAS [112] or EGFR [113]. For example, Tseng’s group released tumor cells from the blood samples of non-small cell lung cancer patients (NSCLC) by the thermo-responsive NanoVelcro platform and detected the mutation of the EGFR gene (confirmed by tissue-based PCR and Sanger sequencing). They found that the L858R or T790M point mutations of the EGFR gene existed and were consistent with the matching tumor tissues. Through the follow-up observation of the patients after drug treatment (EGFR inhibitor, gefitinib), it was found that the evolution of EGFR mutation is related to drug resistance (Figure 7B) [114]. This CTC-based mutational analysis approach has potential value for monitoring cancer evolution and guiding the implementation of targeted therapy.

WGA techniques can produce sufficient genetic material, allowing the detection of genomic alterations at single-cell level [115,116]. Zhao et al. used the PNNanoVelcro chip and laser capture microdissection assay to isolate single advanced prostate cancer CTCs for whole-exome analysis (WES) [117]. They achieved 30× depth and ≥95% coverage. In addition, the shared genomic alterations between CTCs and the matching tumor tissues were identified through single CTC WGA. WGA techniques help us to study the heterogeneity of CTCs and characterize the biological evolution of cancer.

### 3.3. CTC Transcriptomic Profiling

In addition to genome sequencing, RNA sequencing of transcriptome profiling has been widely used in cancer research and contributes to the analysis of tumor biology [118]. Kozminsky et al. used a microfluidic graphene oxide nanoroughened structure-based device (GO chip) to isolate CTCs and CTC clusters from the whole blood of metastatic castration-resistant prostate cancer patients [83]. The principle of this method has been described in Section 2.2.2. Additionally, the cell isolation technology coupled with reverse-transcription polymerase chain reaction (RT-qPCR) was used to detect cancer-specific genes (e.g., AR-FL, AR-V7, KLK3, FOLH1) and RNA (SChLAP1) of prostate CTCs. They found that the levels of these target molecules were significantly increased in patients with metastatic diseases.

**Figure 8:**
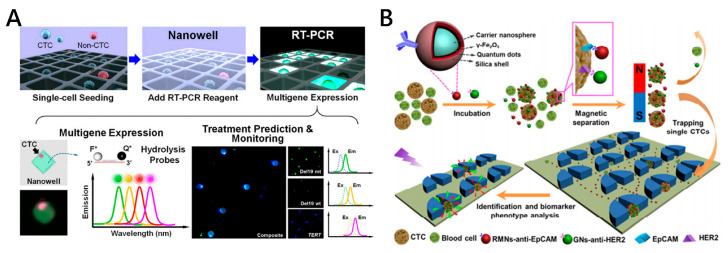
(**A**) Multigene expression profiling of individual CTCs using combined magnetic sifter and nanowell array. Reproduced with permission from [119]; (**B**) Schematic diagram for single-cell biomarker analysis of heterogeneous CTCs using multifunctional nanospheres integrated with micro-array chip. Reproduced with permission from [122].

In order to achieve high throughput of single-cell RNA sequencing (scRNA-seq), there have been parallel multigene expression profiling methods developed. Park et al. reported a platform integrating the magnetic nanoparticles modified with anti-EpCAM antibodies and a magnetic sifter structure for multigene expression profiling of individual CTCs in parallel from NSCLC patients (Figure 8A) [119]. The captured cells are scattered in the nanowell of a magnetic sieve, which provides an isolated environment for cells, allowing for easy parallel detection. In addition, Cheng et al. proposed a Hydro-Seq platform for parallel scRNA-seq which was composed of 800 chambers per chip to accommodate CTCs from 10 mL of patient blood. By pairing single-barcode beads with single cells in microchambers, mRNA molecules from a single cell can be uniquely labeled by a barcode and identified using scRNA-seq [120]. Their work provides a high-throughput and versatile single-cell transcriptomic profiling strategy toward non-invasive cancer diagnosis and monitoring.

### 3.4. CTC Protein Analysis

Single-cell protein analysis plays an important role in biomarker discovery, disease diagnosis, pathology and treatment [121]. CTCs are heterogeneous in phenotype. The discovery of some carcinogenic specific proteins of CTCs can provide a new direction for early diagnosis of diseases. Traditional proteomic methods, such as Western blotting, enzyme-linked immunosorbent assay and mass spectrometry, require a large amount of sample and are not suitable for liquid biopsy-based CTC analysis, masking the heterogeneity of single cells. Currently, researchers conduct cell protein analysis by relying on labeling the target proteins with fluorescent tags. Nanomaterials with unique magnetic or optical properties have been fabricated for multiplex protein analysis of single CTCs [122,123,124]. Pang’s group proposed a micropillar chip-assisted multifunctional-nanosphere system for the capture and analysis of biomarker phenotypes of individual CTCs (Figure 8B) [122]. Red fluorescent magnetic nanospheres modifying anti-EpCAM antibody and green fluorescent nanospheres modifying anti-HER2 antibody were targeted to two kinds of CTC biomarkers, endowing simultaneous magnetic labeling and fluorescence measurement. After trapping CTCs by size-selective chip, easy-readout fluorescence signals from attached nanospheres offered reliable single-cell phenotype analysis and guiding of personalized anticancer therapy. Zhang et al. fabricated multiple surface-enhanced Raman scattering (SERS) bioprobes for the in situ profiling of cell membrane proteins and identification of cancer subpopulations [123]. Three kinds of spectrally orthogonal SERS aptamer nanovectors are designed, providing individual cells with composite spectral signatures in accordance with surface protein expression. Combined with categorization algorithm, cells from different human breast cancer subtypes can be reliably classified with high sensitivity and selectivity. Except for SERS-based protein analysis, surface plasmonic resonance (SPR) technology with the advantages of label-free and high-sensitivity also provides a new method for the detection and analysis of tumor markers of captured tumor cells. Law et al. developed a nanoparticle-enhanced SPR biosensor for detecting tumor necrosis factor alpha antigen with sensitivity enhancement [124].

### 3.5. CTC Functional Profiling

The current research for CTC functional profiling promotes our understanding of CTC phenotype characteristics and their impact on tumor metastasis. The primary culture of CTCs shows their special metabolic characteristics, which is helpful in exploring the functional properties of tumor cells, including migration, adhesion, metabolism and drug response [125,126]. Microfluidic technology bears remarkable advantages in providing real-time monitoring and simulative microenvironments, and the combination with nanotechnology also provides some new strategies for CTC functional profiling. Tumor metabolism has a special characteristic, namely the “Warburg effect” [127]—that is, even in the presence of oxygen, tumor cells prefer to produce energy through the glycolysis pathway. Due to the low energy efficiency of glycolysis, tumor cells exhibit enhanced uptake of glucose [128,129]. Zhang et al. proposed a nanowell chip which contains thousands of nanoliter grooves and allows imaging by fluorescent glucose analogues to assess the glucose uptake of individual CTCs [130]. The nanowell device realizes the multidimensional molecular analysis with minimum cell loss (<20%). They found that tumor cells harboring mutant EGFR exhibited greater glucose uptake than those possessing wild-type EGFR. The metabolism of tumor cells may be related to the activation or inhibition of specific cancer genes. Kelley’s group proposed a magnetic nanoparticle-mediated microfluidic system to capture CTCs and investigate the metabolic activity of different CTC subpopulations [131]. They found that tumor cells with different expression levels of EpCAM show different metabolic responses, such as collagen uptake. The results suggested that metabolism profiling could be referenced to monitor the phenotypic changes of CTCs and to assess their invasion ability. Yao et al. investigated the short-term viability, invasiveness and secretory profiles of individual CTCs using a regular array of nanowells, with the convenience of easy positioning of the nanowell’s location (so called “spatially addressable array”) [132]. CTCs were tracked by recording the changes in the CTC position to characterize their invasion ability. The results showed that only a rare subset of CTCs possessed malignant traits. They also found that single CTCs and CTC clusters from the same patient exhibited obviously different invasive behaviors.

The challenges of analyzing the functional characteristics of CTCs at present are the unsatisfactory viability of released CTCs and the difficulty of conducting continuous cultivation in vitro, so most of the research is still limited to the analysis of tumor cell lines rather than individual CTCs. In addition, the correlation between CTC function and molecular mechanism (genotype and phenotype) remains worthy of further exploration.

## 4. Conclusions

Microfluidics, especially in combination with mechanics, electromagnetics, acoustics and biology, provides efficient approaches to cell isolation and manipulation. Each method has respective strengths and weaknesses depending on the downstream application. Microfluidics has become increasingly integrated and shows high sensitivity and high throughput. Recent developments and applications of captured CTCs have been used to explore cancer mechanisms and address increasingly complex biological questions. There are a variety of CTC isolation and analysis methods which been proposed by far. Nevertheless, most of the technologies are still in the stage of laboratory research; it is still a challenge for these techniques to develop into routine clinical practice. CTC capture still has some challenges, such as the clogging problem based on the microfluidic environment, EpCAM low-expression CTC loss based on the cell affinity method and cell damage caused by microfluidic shear forces. In order to promote clinical application of CTCs, specific adhesive biomolecules, creative device structure designs and smart biomaterials might dramatically change the trend of CTC acquisition in the future. A portable, easy to use and inexpensive system can significantly influence early stage diagnosis, therapy monitoring and cell-based therapy.

Releasing the captured CTCs from nanostructures with less invasive and more controllable strategies is still the major challenge in CTC analysis. Although many physical and biochemical methods have been proposed, there are still difficulties in terms of selectivity, efficiency and cell-friendliness. For example, some release methods based on temperature, light, electricity or chemical reagents will introduce external stimuli to cells, thus affecting their original function and viability. Additionally, the specificity of the released cells is not high enough to realize accurate CTC “selection” using existing approaches, so the subsequent genomic or protein profiling analysis will be disturbed. Before extending a single CTC analysis to routine clinical practice, many issues must be solved, including accuracy, reliability, repeatability, cost, etc. In order to promote clinical application of CTCs, specific adhesive biomolecules, creative device structure designs and smart biomaterials might dramatically change the trend of CTC acquisition in the future. A portable, easy to use and inexpensive system can significantly influence early stage diagnosis, therapy monitoring and cell-based therapy.

The purpose of cell capture and release is to perform cell detection and analysis, such as cell culture, single-cell sequencing, protein analysis, metabolism analysis and drug sensitivity experiments, etc. Single-cell analysis is the main direction of downstream operations. For single-cell operations, it is highly desirable to develop single-cell isolation techniques with high sensitivity, accuracy and easy operation. On the other hand, moving forward, advances in biology are expected to initiate the development of new cell process methods. The biological issues underlying these methods may further address a range of single-cell analysis questions via molecular biology assays, on which more research will focus in the future. These new methods will help us to explore the relationship between CTC molecular information and clinical assessment.
